# Prevalence and Associated Factors of Depression Among Elderly Hypertensive Patients in Vietnam

**DOI:** 10.3390/geriatrics10050129

**Published:** 2025-10-01

**Authors:** Tuan Van Nguyen, Tung Son Vu, Thang Thien Tran, Thong Thai Nguyen, Hoang Minh Le, Thang Nguyen, Kha Ai To Tran, Chau Minh Tran, Thong Van Nguyen

**Affiliations:** 1 National Institute of Mental Health, Bach Mai Hospital, Hanoi 100000, Vietnam; nguyenvantuan@hmu.edu.vn; 2 Department of Psychiatry, Hanoi Medical University, Hanoi 100000, Vietnam; vusontung269@gmail.com; 3 Department of Psychiatry, Can Tho University of Medicine and Pharmacy, Can Tho City 900000, Vietnam; ttthang@ctump.edu.vn (T.T.T.); nthaithong@ctump.edu.vn (T.T.N.); 4 Department of Traditional Medicine, Can Tho University of Medicine and Pharmacy, Can Tho City 900000, Vietnam; lmhoang@ctump.edu.vn; 5 Department of Pharmacology and Clinical Pharmacy, Can Tho University of Medicine and Pharmacy, Can Tho City 900000, Vietnam; nthang@ctump.edu.vn; 6 Department of Medicine, Can Tho University of Medicine and Pharmacy, Can Tho City 900000, Vietnam; 2253010103@student.ctump.edu.vn; 7 Department of Public Health, Can Tho University of Medicine and Pharmacy, Can Tho City 900000, Vietnam; tmchau7902@gmail.com

**Keywords:** late-life depression, hypertension, sleep quality, loneliness, Vietnam, inpatients

## Abstract

**Background/Objectives:** Depression accompanying hypertension increases the burden of illness and negatively affects patients’ lives. However, depression among elderly with cardiovascular diseases in general and hypertension in specific has not been paid proper attention, especially in the context of Vietnam. Therefore, we expected to examine the prevalence, characteristics, and related factors of depression on elderly patients with hypertension. **Methods:** A cross-sectional study at the Department of Geriatrics, Can Tho Central General Hospital (from April 2020 to February 2022), involving 414 patients aged ≥60 years with hypertension. Depression was assessed using ICD-10 diagnostic criteria; related factors were evaluated using the Pittsburgh Sleep Quality Index (PSQI), KATZ12 index, and UCLA-LS3-J11 scale. **Results:** 31.4% of participants were found to have depression (mild 17.7%, moderate 9.7%, severe 4.0%). Common characteristics of depression included sleep disturbances, decreased energy, and low mood. Poor sleep quality, being female, lower socioeconomic status, higher hypertension grade, and increased loneliness were found to be significantly associated with depression. **Conclusions:** The high prevalence of depression among elderly hypertensive patients highlights the need for effective screening and intervention strategies. Addressing factors such as sleep quality, gender, socioeconomic challenges, and social isolation may help mitigate the burden of depression in this vulnerable population. This research should be expanded to elderly patients with hypertension in society, outpatients, and individuals with other chronic diseases. Developing a predictive model for depression in elderly patients, particularly those with chronic diseases, can improve early detection, treatment effectiveness, and overall care quality.

## 1. Introduction

The number and proportion of people aged 60 years and older in the population is increasing, and the elderly have become the point of new action on aging and health issues [1]. According to United Nations Population Fund (UNPFA), Vietnam is among the countries with the fastest aging populations in the world. Individuals aged 60 years and older accounted for 11.9% of the total population in 2019, and this figure is projected to rise to over 25% by 2050 [2]. An ageing population tends to have a higher prevalence of chronic diseases, physical disabilities, mental illnesses and other co-morbidities [3].

Hypertension already affects an estimated 1.28 billion adults and causes about 10.8 million deaths each year, remaining the leading preventable cardiovascular risk worldwide [4]. The prevalence of hypertension increases with age; in the older adults over 60 years of age, it exceeds 60%, significantly impacting both health status and quality of life [4]. Around 14% of adults aged 60 and over live with a mental disorder, with depression being one of the most common [5]. Late-life depression is diagnosed in roughly 12–17% of community-dwelling older adults and is 1.5- to 2-times more common in those with hypertension, according to recent meta-analyses encompassing more than 100,000 participants [6]. Despite this dual burden, up to 60% of depressive episodes in hypertensive seniors go unrecognised in primary care and only one-third receive guideline-concordant treatment [7,8]. Detection is hampered because older patients often present “masked” or somatic symptoms—fatigue, pain, disturbed sleep—rather than overt sadness, leading clinicians to attribute complaints to cardiovascular disease or ageing itself [9]. Cohort and hospital studies additionally show that longer hypertension duration, higher blood-pressure grade, poor BP control, and multimorbidity each raise the odds of comorbid depression, whereas regular physical activity and good perceived health are protective [10,11,12].

Although previous studies have examined the link between hypertension and depression, few have focused on elderly inpatients in lower–middle-income countries like Vietnam. Key factors such as sleep disturbance, loneliness, functional dependence, and hypertension severity remain underexplored in this context, where mental health services for older adults are still limited. Moreover, existing studies in Vietnam have largely relied on self-reported measures and mainly focused on people living in the community, resulting in inconsistencies in diagnostic approaches and limited generalizability to clinical settings [13,14,15,16]. These methodological differences pose challenges in accurately identifying depression among older adults, particularly those with comorbid chronic conditions, such as hypertension. In this study, we specifically focused on inpatients with hypertension—a group at high risk both physically and psychosocially. We conducted assessments using the ICD-10 diagnostic criteria to identify depression, thereby providing an accurate estimate of depression prevalence in these patients. A clearer understanding of these relationships will inform targeted screening and integrated management strategies for primary-care settings in Vietnam, where rapid population ageing is converging with high rates of uncontrolled hypertension and under-detected late-life depression. The study aimed to provide more reliable data on the prevalence, characterise the clinical manifestations of depression and to identify the demographic, clinical, and haemodynamic factors most strongly associated with depressive morbidity in Vietnamese adults aged ≥60 years who have hypertension. This study addresses evidence gaps in Vietnam by focusing on elderly inpatients—a clinically vulnerable group—and by applying ICD-10 diagnostic interviews rather than relying solely on self-report symptom scales. We also evaluate under-studied correlates—sleep disturbance, loneliness, and hypertension severity—to inform feasible screening and integrated care pathways in resource-constrained settings.

## 2. Materials and Methods

### 2.1. Study Design and Participants

This cross-sectional study was conducted at the Department of Geriatrics, Can Tho Central General Hospital, from April 2020 to February 2022. Participants included patients aged 60 years and older diagnosed with hypertension (systolic blood pressure ≥ 140 mmHg and/or diastolic blood pressure ≥ 90 mmHg, according to WHO criteria [17]), who were currently undergoing treatment and consented to participate. Patients experiencing acute illnesses limiting communication, language barriers, or severe personal problems within the previous two weeks were excluded.

The required sample size was calculated using the formula:

$$n={\left(Z_{1-\alpha /2}\right)}^{2}\times \frac{p\times (1-p)}{d^{2}}$$

with *Z*_1−*α*/2_ = 1.96, margin of error *d* = 0.05, and estimated depression prevalence *p* = 32.8%, based on Chuan Zou et al. [18], yielding a minimum sample size of 339. Considering a 10% potential dropout rate, the final sample required was 374. Ultimately, 414 patients were included.

### 2.2. Data Collection Process

**Figure 1:**
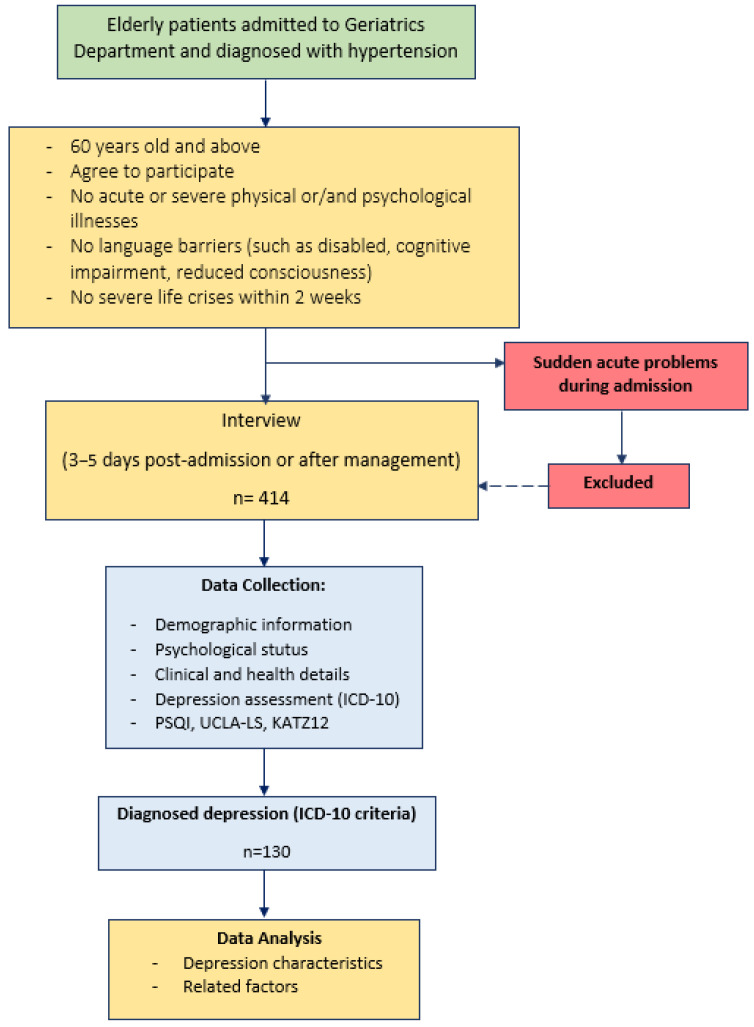
Data collection process.

Participants who met inclusion criteria were interviewed directly to collect demographic, clinical, and medical history data. Interviews were scheduled conveniently to minimize disruption to meals, rest periods, or medication schedules, typically between 2:00–5:00 p.m. or 7:00–9:00 p.m. The data collection procedure is illustrated in Figure 1.

### 2.3. Variables and Measures

Depression was assessed using ICD-10 diagnostic criteria [19], which include the following: characteristic symptoms: (1) low mood, (2) reduced interest or pleasure, and (3) decreased energy or increased fatigue; common symptoms: (1) reduced concentration, (2) diminished self-esteem, (3) feelings of guilt, (4) pessimistic views about the future, (5) suicidal thoughts or behavior, (6) sleep disturbances, and (7) appetite or weight changes. Diagnosis required at least two characteristic symptoms and two common symptoms lasting a minimum of two weeks.

Associated factors were categorized into two groups: socio-demographic factors: age, sex, educational level, living area, socioeconomic status, and living partner; health-related and lifestyle factors: Body Mass Index (BMI) according to Asian adult standards. Associated chronic diseases (e.g., endocrine disorders, respiratory diseases). Hypertension grade was based on Vietnam Heart Association’s guidelines [20]. Hypertension complications identified during the examination or previously diagnosed (heart, brain, eye, kidney complications). Physical activity level classified as low (minimal or no activity), medium (walking/jogging ≤ 16 km/week), and high (walking/jogging > 16 km/week). Sleep quality measured using the Pittsburgh Sleep Quality Index (PSQI) [21], with scores ≥ 5 indicating sleep disturbance. Loneliness evaluated using the UCLA-LS3-J11 scale [22], ranging from 20 to 80, where higher scores reflect greater loneliness. Daily functional independence assessed with the Katz Index of Independence in Activities of Daily Living (KATZ12 index) [23], categorizing functional status as good (5–6 points), moderate (3–4 points), or severely impaired (≤2 points).

### 2.4. Data Analysis

Statistical analyses were performed using SPSS version 20.0. Continuous variables were presented as mean ± standard deviation (SD) (or median [IQR] if skewed), and categorical variables as frequencies and percentages (n and %). Between-group comparisons used independent *t*-tests for normally distributed continuous variables and Mann–Whitney U tests otherwise; for >2 groups we used ANOVA or Kruskal–Wallis as appropriate. Associations between categorical variables were examined with χ^2^ tests (or Fisher’s exact when expected counts < 5).

Factors with *p* < 0.20 in univariable analyses were entered into a multivariable logistic regression (backward stepwise) to identify independent correlates of depression. We report adjusted odds ratios (aOR) with 95% CIs and *p*-values; statistical significance was set at *p* < 0.05. For PSQI and UCLA-LS3-J11 (continuous), aORs reflect the change per 1-point increase. Analyses were conducted in SPSS v20.0.

### 2.5. Research Ethics

This study was approved by the Medical Ethics Committee of Hanoi University, No: 72/GCN-HHĐĐNCYSH-ĐHYHN. All patients were clearly explained about the study and voluntarily participated. During the data collection, if depression was identified, and/or there were negative thoughts or behaviors among the patients, we arranged for a safe and comprehensive treatment for the patient.

## 3. Results

### 3.1. Characteristics of Research Population

Participants had a mean age of 74.92 ± 8.35 years and were predominantly female (68.8%), with education levels mostly below high school. The majority lived in rural areas (73.2%) and reported non-poor economic status. Most had normal BMI and two or fewer additional chronic diseases (64.5%). Hypertension grades were evenly distributed across Grade 1 to Grade 3, while hypertension-related complications were present in approximately 30% of the participants (Table 1).

### 3.2. Prevalence and Characteristics of Depression in Elderly Patients with Hypertension

**Figure 2:**
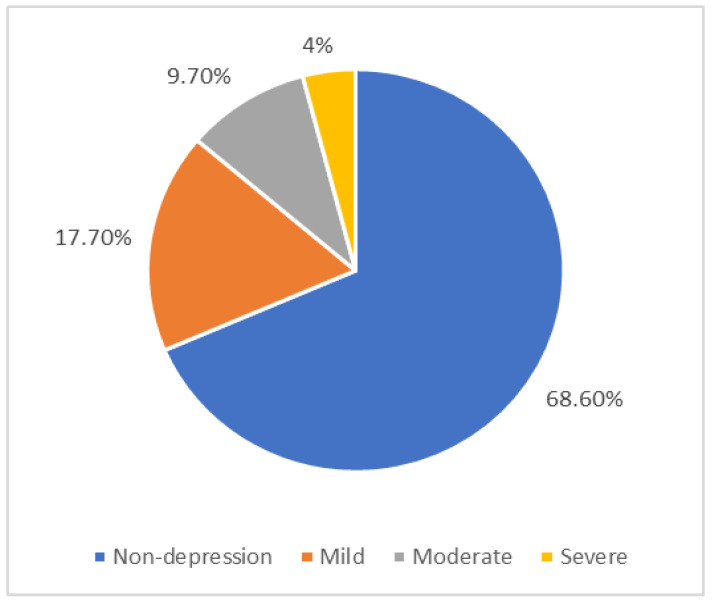
Prevalence of depression and level of depressive disorder.

Figure 2 illustrates the prevalence and severity of depression among study participants. Nearly one-third of participants exhibited depressive symptoms (31.4%), with varying levels of severity.

**Figure 3:**
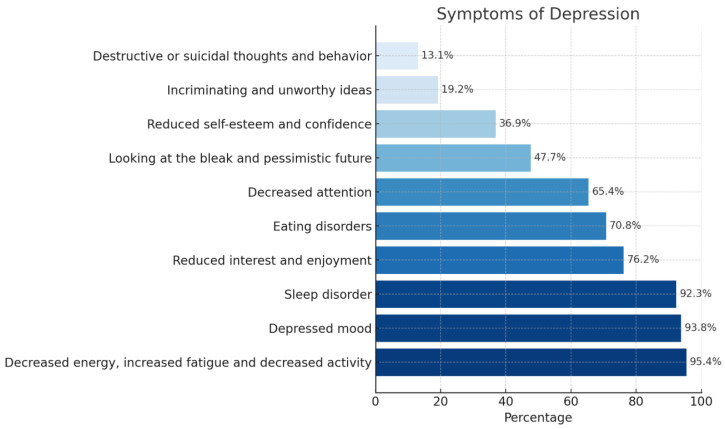
Characteristics of depression according to ICD-10 (*n* = 130).

The predominant depressive symptoms reported were decreased energy, fatigue, and reduced activity, depressed mood, and sleep disturbances. Other frequently observed symptoms included diminished interest or enjoyment, eating disturbances, and impaired concentration. In contrast, psychological symptoms such as pessimistic outlook, low self-esteem, guilt or feelings of worthlessness and suicidal ideation or behaviors were less commonly noted (Figure 3).

### 3.3. Related Factors to Depression in Elderly Patients with Hypertension

We divided the factors related to depression into two main groups: sociodemographic factors, and physical illnesses and life activities factors. For the sociological factors, we did not find a significant difference (*p* > 0.05) in the mean age between the group with depression and the non-depressed group. The prevalence of depression was higher among females (38.6%), individuals with education below high school level (33.6%), and those with lower socioeconomic status (63.3%), with statistically significant differences (*p* < 0.05). For the physical illnesses and life activities factors, including physical illnesses and hypertension, there was a statistically significant correlation (*p* < 0.05) between depression and the degree of hypertension, level of physical activity, PSQI, UCLA score, and KATZ score.

In univariable analyses, female sex, lower socioeconomic status, higher hypertension stage, lower physical activity, poorer sleep quality, greater loneliness, and lower Katz scores were associated with depression. In the multivariable model, female sex, poor socioeconomic status, hypertension stage 2 and 3/ISH (vs. stage 1), higher PSQI, and higher UCLA-LS3-J11 remained independently associated with depression, whereas educational level was not significant after adjustment (Table 2).

## 4. Discussion

### 4.1. Prevalence and Characteristics of Depression: Interpretation and Comparison

#### 4.1.1. Prevalence of Depression

This study found a high depression rate (31.4%) among hypertensive elderly patients, comprising 17.7% with mild depression, 9.7% with moderate depression, and 4% with severe depression. Our result is comparable to the 32.8% depression rate reported in elderly inpatients by Chuan Zou et al. [18], which was used as the reference value for our sample size calculation. Depression in the older age population has been reported in many studies [24,25,26]. Specifically, elderly patients with hypertension are at higher risk of depression than those without [5,27,28]. Prevalence differences across studies might reflect variability in patient settings (community, outpatient, inpatient), with hospitalized patients potentially experiencing heightened depression due to additional stressors such as healthcare costs and uncertainty about health outcomes. Hospitalized patients may endure more risk of depression due to concerns about health care costs, length of stay, and outcomes [29]. These findings indicate that depression should be given more attention in the elderly hypertensive populations and underscore the necessity of routine depression screening and targeted care interventions for these patients.

#### 4.1.2. Clinical Characteristics of Depression

Decreased energy, depressed mood, and sleep disturbances were identified as prominent depressive symptoms. Depression presentations in older adults typically differ from younger populations; cognitive–emotional symptoms like irritability and guilt are less commonly reported in the elderly, whereas somatic symptoms such as fatigue, psychomotor slowing, and sleep disturbances predominate [30,31]. There appears to be a relationship between depressive symptoms and hypertension. Sleep disturbances are a common symptom, often found in elderly patients with depression [32,33]. Thus, depressive symptoms in elderly individuals with hypertension can also be easily mistaken for symptoms and consequences of physical illnesses. Clinicians should maintain high suspicion of depression when hypertensive elderly patients present with these somatic symptoms, regardless of physical disease control status.

In our study, the use of ICD-10 diagnostic criteria to assess depression may improve clinical accuracy and reduce potential bias from participant self-assessment, especially given that depressive symptoms in older adults often present in somatic forms that may be mistaken for physical health problems.

Distinguishing between “depressive symptoms” and a formal “diagnosis of depression” is necessary in mental health research among the elderly, particularly in populations with chronic conditions. The use of standardized diagnostic criteria may help ensure more consistent identification and offer more clinically relevant insights.

### 4.2. Factors Associated with Depression: Analysis and Implications

#### 4.2.1. Sociodemographic Factors

The prevalence of depression in females (38.6%) was higher than in males (15.5%), with statistically significant differences. Many studies also indicate gender differences in depression rates, with females being more at risk [28,34,35,36,37]. This difference could be due to differences in coping style [38] and hormone change [39] in late-life women. Economic areas were also a significant factor related to depression. Poor socioeconomic status groups (63.3%) had a higher prevalence of depression compared to non-poor groups (28.9%), aligns with the study by Ashok et al. (2019), who reported similar associations between lower socioeconomic status and higher depression prevalence [40]. Moreover, contrary to most international findings showing higher depression prevalence in urban areas [41,42,43], this study observed higher depression rates in rural populations. In the context of Vietnam and some other developing countries, this difference is due to the variations in marital status, ethnicity, and socioeconomic status between societies. Patients living in rural areas may visit hospitals infrequently, receive less outpatient treatment, and lack education and mental health care services [44]. Furthermore, urbanization in developing countries led to a movement of the younger population to urban areas and most of the elderly were left to look after themselves in the rural areas.

#### 4.2.2. Physical Illnesses and Life Activities Factors

Severity of hypertension, loneliness, and sleep quality are significantly associated with depression. The average loneliness score (UCLA-3) in the depressed group (43.02) is higher than in the non-depressed group (31.57). Elderly patients tend to be more vulnerable to loneliness due to life changes and losses. Loneliness was emphasized as a significant risk factor for depression and hypertension progression among older adults [5,45]. A decrease in interests and activities among hypertensive elderly patients can increase the feeling of loneliness, hence, increasing the rate of depression. In addition, in Vietnamese culture, there is a strong expectation that younger family members will take care of the elderly. When this family support is absent or limited, older adults may feel neglected or lonely, which can increase their risk of depression [15]. This has become even clearer as Vietnam undergoes continued development, urbanization, and modernization. These cultural and social factors may partly explain why depression is more common in this group.

Depression prevalence was higher among individuals with more severe hypertension, consistent with previous findings linking depression severity to hypertension grade [28,46]. There is a strong link between depression, sleep disorders, and hypertension. Poor sleep quality was prevalent in 92.3% of depressed participants, highlighting a bidirectional relationship between depression and sleep disturbances. Sleep disorders may not only be symptomatic of depression but can also contribute to its worsening [32,47,48]. On the other hand, sleep disorders affected hypertension status, which in turn caused physical and mental discomfort as well as poor sleep quality [49]. This relationship may be further complicated by the use of antihypertensive medications, which have also been significantly associated with poor sleep status in hypertensive individuals [50]. Therefore, effective management of sleep problems could have significant implications for improving outcomes related to both hypertension and depression in older adults.

### 4.3. Limitations and Recommendations

During data collection, the COVID-19 pandemic in Vietnam led to a shift in patient structure due to admission restrictions at Can Tho Central General Hospital. Additionally, patients from other provinces faced difficulties in transferring to Can Tho for hospital admission in the background of the pandemic outbreak. The pandemic itself may have also influenced participants’ mental health, potentially elevating levels of anxiety, loneliness, or depression beyond their typical baseline. Fatigue and limited concentration among patients during the interviews may have compromised the accuracy and reliability of the data obtained. This study could be extended to include patients in community, outpatients, or patients with other chronic diseases, such as endocrine disorders, respiratory diseases, and musculoskeletal diseases. This can support developing a comprehensive predictive model for depression in elderly patients. The model would enable early identification and intervention for depression in the aging population, thereby enhancing the overall outcomes and reducing the burden of depression on the elderly patients with chronic diseases. Furthermore, it could also lead to more effective treatment plans, resulting in better physical and mental quality in health care.

## 5. Conclusions

Approximately one-third of elderly hypertensive patients experienced depression. These findings underscore the necessity of developing a screening plan for depression in this vulnerable group. Factors such as sleep quality, gender, socioeconomic status, hypertension grade, and loneliness were found to have a significant association with depression in these patients. It is important to pay attention to signs of depression when elderly patients have physical complaints such as fatigue, decreased activity, and sleep disturbances, even if physical factors are reasonably controlled. Effective screening and treatment of depression alongside managing hypertension can improve patient outcomes.

In the context of Vietnam, where the population is rapidly aging and the burden of chronic non-communicable diseases is increasing, these findings are particularly relevant. Including depression screening as part of routine care for older hypertensive patients at the primary healthcare level may be a cost-effective and practical solution to help close this gap. In addition, community-based approaches that are adapted to the local culture and social environment are necessary to improve early detection of depression and enhance the quality of life for older adults.

Moreover, this research could expand to elderly patients in community, outpatients, and individuals with other chronic conditions. Developing a comprehensive predictive model is recommended to enhance early detection and targeted intervention strategies for depression in elderly populations.

## Figures and Tables

**Table 1:** Characteristics of participants.

Characteristic		Frequency (*n*)	Ratio (%)
Age	Youngest: 60, Oldest: 101 Mean: 74.92 (Standard deviation: 8.35 years old)		
Sex	Female	285	68.8
	Male	129	31.2
Level Education	Below high school	372	89.9
	High school and higher	42	10.1
Status marriage	Single/divorced/separated/lost spouse	147	35.5
	Have a spouse living together	267	64.5
Economy society	Poor	30	7.2
	Not poor	384	92.8
Living area	Rural areas	303	73.2
	Urban areas	111	26.8
BMI	Underweight	49	11.8
	Normal	310	74.9
	Overweight—Obesity	55	13.3
Number of other physical diseases	>2	147	35.5
	2 and under	267	64.5
Grade of hypertension	Grade 1	141	34.1
	Grade 2	140	33.8
	Grade 3 and Isolated systolic hypertension	133	32.1
Hypertension complication	Have	123	29.7
	None	291	70.3

**Table 2:** Factors associated with depression (ICD-10) in elderly inpatients with hypertension (*n* = 414).

Factor	Category/Unit	Depressed *n*/N (%)	Unadjusted *p*	Adjusted OR (95% CI)	Adjusted *p*
Gender	Male vs. Female (ref)	20/129 (15.5) vs. 110/285 (38.6)	<0.001 (χ^2^)	0.35 (0.18–0.68)	0.002
Socioeconomic status (SES)	Not-poor vs. Poor (ref)	111/384 (28.9) vs. 19/30 (63.3)	<0.001 (χ^2^)	0.33 (0.12–0.91)	0.032
Living area	Urban vs. Rural (ref)	29/111 (26.1) vs. 101/303 (33.3)	0.162 (χ^2^)	0.69 (0.36–1.31)	0.256
Comorbidities	≤2 vs. >2 (ref)	83/291 (28.5) vs. 47/123 (38.2)	0.052 (χ^2^)	0.72 (0.40–1.30)	0.278
Hypertension stage	2 vs. 1 (ref)	49/140 (35.0) vs. 33/141 (23.4)	0.041 (χ^2^)	2.02 (1.03–3.96)	0.041
	3/ISH vs. 1 (ref)	48/133 (36.1) vs. 33/141 (23.4)	—	2.38 (1.20–4.73)	0.014
Physical activity	Medium vs. Low (ref)	38/200 (19.0) vs. 90/200 (45.3)	<0.001 (χ^2^)	0.61 (0.33–1.10)	0.099
	High vs. Low (ref)	1/13 (7.7) vs. 90/200 (45.3)	—	0.89 (0.08–9.51)	0.926
PSQI	per 1-point increase	mean 12.7 ± 4.2 vs. 8.4 ± 4.0	<0.001 (Mann–Whitney U)	1.22 (1.14–1.31)	<0.001
UCLA-LS3-J11	per 1-point increase	mean 43.0 ± 12.3 vs. 31.0 ± 9.4	<0.001 (Mann–Whitney U)	1.07 (1.05–1.10)	<0.001
Katz	per 1-point increase	mean 4.65 ± 1.91 vs. 5.38 ± 1.40	<0.001 (Mann–Whitney U)	0.92 (0.78–1.09)	0.325

Notes: Unadjusted *p* from χ^2^ (categorical) or Mann–Whitney U (continuous). Adjusted OR and *p* from multivariable logistic regression including gender, SES, living area, hypertension stage, physical activity, PSQI, UCLA-LS3-J11, and Katz. Ref = reference category; ISH = isolated systolic hypertension.

## Data Availability

The original contributions presented in this study are included in the article. Further inquiries can be directed to the corresponding author.

## Abbreviations

The following abbreviations are used in this manuscript:

WHO	World Health Organization
BMI	Body Max Index
